# Understanding the Impact of Individual Nucleotide on Oxford Nanopore Current Signals With Interpretable Prediction Models

**DOI:** 10.1177/11779322251378620

**Published:** 2025-09-22

**Authors:** Yenan Wang, Zhixing Wu, Jia Meng

**Affiliations:** 1 Department of Biosciences and Bioinformatics, Center for Intelligent RNA Therapeutics, Suzhou Key Laboratory of Cancer Biology and Chronic Disease, School of Science, Suzhou, China; 2 School of Biosciences, Faculty of Science, University of Melbourne, Parkville, VIC, Australia; 3 School of AI and Advanced Computing, XJTLU Entrepreneur College, Xi’an Jiaotong-Liverpool University, Suzhou, China; 4 Department of Computer Science, University of Liverpool, Liverpool, UK; 5 Institute of Systems, Molecular and Integrative Biology, University of Liverpool, Liverpool, UK

**Keywords:** Oxford nanopore, current signal, DNA, sequencing, prediction, machine learning

## Abstract

Oxford nanopore sequencing enabled real-time, long-read analysis of DNA by detecting ionic current signals associated with K-mer sequences. Although many studies analyzed sequence and modification detection, our understanding of how multiple nucleotides of the K-mer sequence determine nanopore signals together is still limited. In this study, we seek to unveil the positional impact of individual nucleotide through interpretable prediction models. Multiple machine learning models were trained and optimized. To increase model interpretability and explore underlying mechanisms, the tool of SHapley Additive exPlanations was applied to make an assessment of both nucleotides and positions. Our results show that previously unseen Oxford nanopore signals were accurately predicted, and results were consistent on two different modes (R^2^ = 0.9984 for 260 bps, R^2^ = 0.9983 for 400 bps, R10.4 flow cell, XGBoost). Thymine bases (T) at positions 6 and 7 were the most influential, while nucleotides at positions 1, 2, 3, 4, and 9 have minimal impacts on signals. In addition, heatmap analysis toward transitions of bases revealed the impact of individual nucleotide on signal changes in a position-specific manner. Briefly, our work provided predictive and interpretable modeling of nanopore signals, concentrating on influential bases and positions among all obtainable features, which enhanced understanding of nanopore sequencing mechanisms and nucleotide/position-related signal variations.

## Introduction

Nanopore sequencing has revolutionized the field of genomics by enabling real-time, long-read sequencing of DNA and RNA molecules.^1-3^ Unlike traditional short-read sequencing methods, nanopore sequencing directly detects nucleotide sequences by measuring changes in ionic current as nucleotides pass through a biological nanopore.^4,5^ This technology has been widely applied in various areas such as genome assembly, modification detection, transcriptomics, and epigenetics,^2,6,7^ while the detailed mechanism of nanopore signal changes and potential connection with information of sequence positions and bases have still belonged to a significant and challenging research topic due to the complex and stochastic nature of current fluctuations and base effects.^2,8^

In order to dive into such field, various computational approaches have been employed for nanopore signal analysis. Hidden Markov Models (HMMs) were among the earliest methods used to decode electrical signals into nucleotide sequences.^9^ Later, deep learning (DL) models such as Recurrent Neural Networks (RNNs) and Convolutional Neural Networks (CNNs), demonstrated superior performance in base calling and modification detection.^10,11^ Machine learning (ML) techniques, including support vector machines (SVMs) and random forests (RF), have also been explored for detecting modified nucleotides from nanopore sequencing data.^12^

Despite the success of various models, there appears to be a lack of robust, interpretable models applied to nanopore sequencing analysis with predictive purposes.^2^ Specifically, there seems to be a lack of a well-established series of models consistently used for signal prediction, since many existing models lack interpretability,^13^ making it difficult to understand the true relationship between nucleotide sequences and signal variation.

In addition, previous studies have primarily focused on 5-mer sequences, while analysis of longer sequence length seems to be a blank field.^5,14^ It is evident that the studies of 9-mer DNA sequences with the inclusion of nine nucleotides through the nanopore could be rather meaningful and practical by increasing the input dimensionality and capturing more contextual information.^14^ This expansion might introduce greater complexity but has also provided a richer representation of sequence context, potentially enhancing the resolution of signal interpretation in nanopore data.

In this study, we would like to explain the mechanism between nanopore bases, positions, and the ionic current values, especially enabling the predictive ionic analysis based on the 9-mer DNA sequences. Specifically, a series of ML and DL models could be put forward with comparisons in order to find the optimal tools with outperformed metrics. To improve model transparency, effective tools would also be employed to interpret feature contributions, revealing position-specific nucleotide effects on the current signal. In addition, we expect to incorporate heatmap analyses to visualize base-to-base transitions across all positions in 9-mer sequences, aiming to identify potential patterns of signal variation associated with nucleotide changes. Therefore, our novel investigations would contribute to the growing body of work on interpretable predictive models for nanopore sequencing, explore influential effects from either positional or nucleotide-related features, and further help bridge the gap between advanced learning methods and base-level signal interpretations.

## Method

### Nanopore sequencing reference signals

The dataset used in this study is from the publicly available 9-mer DNA sequence database by Oxford Nanopore Technologies (ONT) Ltd.^1^ This database, part of Oxford Nanopore’s Open Data Initiative, supports research on nanopore signal characteristics and computational analysis tools.^1,15^ Numerous prior studies have leveraged ONT datasets to benchmark base calling algorithms and improve signal-processing methods, particularly in the context of long-read sequencing.^11,16,17^ Its accessibility and comprehensive coverage make it a reliable foundation for modeling the relationship between nucleotide sequences and ionic current signals.

The data were normalized to ensure consistency, with target variables standardized (zero mean and unit variance) for comparability. K-mer level tables, which represent sub-sequences of the DNA consisting of “K” nucleotides,^18^ were used to estimate the expected signal levels for each K-mer, improving the accuracy of detecting modified bases. These tables have been standardized with a mean of zero and standard deviation of one and were further organized by sequencing conditions.

After data preprocessing to ensure the absence of missing values, we confirmed that nucleotide frequencies are evenly distributed across all positions. Each 9-mer sequence consists of 9 positions, each with one of four possible nucleotides (A, G, C, T), leading to 262 144 possible combinations. The distribution of these combinations is uniform. The throughput of nanopore sequencing is commonly measured in base pairs per second (bps), indicating the number of nucleotides that pass through the pore per unit time.^15^ Our studies mainly focused on two datasets with both 9-mer DNA sequences, one from 260 bps and the other from 400 bps.

### Predictive and targeted variables

The predictors in this study were derived from the 9-mer DNA sequences to understand the relationship between nucleotide arrangements and electrical current levels. Each sequence is transformed into numerical features using One-hot Encoding, a method that converts categorical variables into binary vectors, with a label of zero denoting absence and a label of one denoting existence, respectively.^19^

Each nucleotide position (out of four possible nucleotides: A, C, G, T) could be represented as a four-dimensional vector for each position. For example, the four nucleotides at the first position (“A, C, G, T”) are encoded as “Position1_A, Position1_C, Position1_G, Position1_T,” and similar transformations are applied to the remaining positions. A total of nine positions together with four nucleotide types were taken into consideration, and such an encoding procedure would result in a combination of 36 features for each of the 9-mer DNA sequences.

For corresponding nanopore ionic current signals, the mean values of electric currents were matched with each of the 9-mer sequences, which were utilized as the observable targets in our predictive model for supervised learning purposes.

### Model comparison and best model selection

To investigate the relationship between nucleotide sequences and electrical current levels in nanopore sequencing, we applied advanced frameworks such as statistical and ML approaches. Several models were tested for their ability to predict electrical signals from 9-mer DNA sequences, including the generalized linear model (GLM), decision tree (DT), gradient boosting, K-nearest neighbors (KNN), SVM, and XGBoost (XGB).

Several key performance metrics are used to assess the model performance. The Coefficient of Determination (R^2^) indicates how well the model explains the variance in the dependent variable, with a range from 0 to 1, where a larger figure denotes a better model fit.^20^ Mean absolute error (MAE) measures the average absolute difference between predicted and actual values, providing a more intuitive error measurement.^21^ In addition, mean squared error (MSE) is usually adopted for measuring the average squared difference, while root mean squared error (RMSE) is another form by taking the square root values.^21^

The dataset has typically followed the 70-30 rule, where 70% of observations were split for training and the remaining 30% for testing purposes and performance evaluation.^22^ To ensure fitting robustness and avoid overfitting problems, k-fold Cross-Validation is used to evaluate the model (where k = 10 in our studies), which rotates validation subsets across multiple iterations.^23^ Specifically in k-fold Cross-Validation, the dataset is divided into k equal-sized parts (or “folds”), where each fold is used once as the validation, with the remaining k−1 folds used for training, and the process is repeated k times to obtain the average performance estimate.^23^ In addition, regularization technique such as L2 regularization (via the lambda parameter in XGB) was applied, while early stopping was used during training using a separate validation set, with a patience of 20 rounds. Moreover, a comprehensive grid search over key hyperparameters (e.g. max_depth, learning_rate, subsample, and colsample_bytree) was performed to fine-tune and optimize model performance.

### ML explainable tool

The ML models are often considered “black boxes” by experts due to their complex internal mechanisms and lack of transparency.^24^ An effective supportive tool called SHapley Additive exPlanations (SHAP) was proposed by Lundberg and Lee,^25^ based on Shapley value theory from cooperative game theory, which could quantify the contribution of each feature to the model’s output, thereby enhancing both explainability and interpretability.

In the context of our DNA nanopore sequencing, SHAP tools could help identify the nucleotide patterns in 9-mer fragments that contribute most to the prediction of electrical current levels. It could also provide clear visualizations that display feature importance, highlighting the factors that influence the model’s predictions.^26^ This effect has successfully assisted in model optimization and improved our understanding of sequence pattern recognition in nanopore signal analysis.^26^

### Heatmap analysis and base change effects

In order to better illustrate the specific effects on ionic current signals in correspondence with nucleotide base changes, heatmap figures have been drawn with different regression models constructed. In these plots, the horizontal x-axis represents the Original base, the vertical y-axis indicates the Target base, and the color gradient reflects the regression coefficients corresponding to the direction and magnitude of ionic signal changes.^27^ The color of red denotes the positive trend of current changes, and the color blue shows the negative, where darker boxes exhibit more evident signal fluctuations due to base substitution. The elements symmetric with respect to the anti-diagonal are additive inverses of each other, showing the opposite direction of signal numbers with the switch of transitioned nucleotides.

## Result

### Model building and evaluation

**Figure 1.**
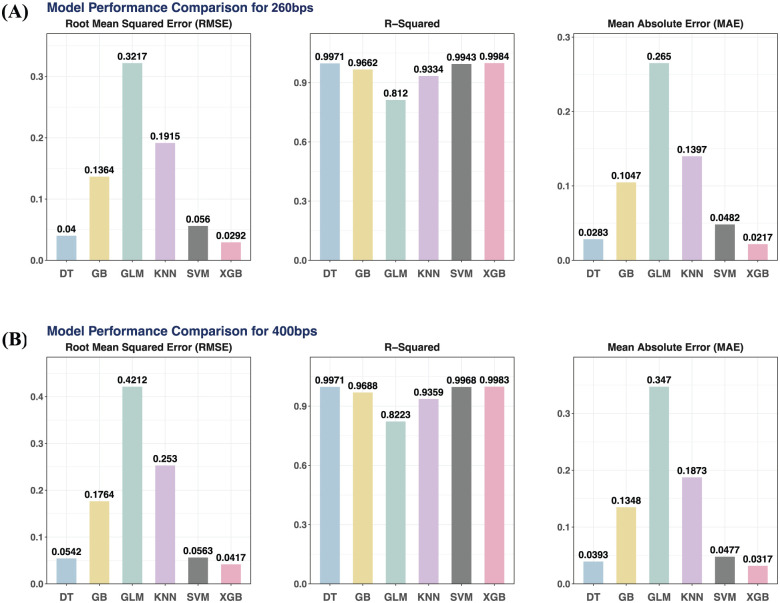
Model performance comparison in terms of RMSE (root mean squared error), R-squared and MAE (mean absolute error) for DNA 9mer ONT Database with 260 bps (A) and 400 bps (B), respectively. DT, decision tree; GB, gradient boosting; GLM, generalized linear model; KNN, K-nearest neighbors; SVM, support vector machine (Radial); XGB, XGBoost.

As illustrated in Figure 1A (260 bps) and Figure 1B (400 bps), XGB consistently outperformed the other models across all three evaluation metrics. It achieved the lowest RMSE values (0.0292 for 260 bps and 0.0417 for 400 bps) and MAE values (0.0217 and 0.0317), while maintaining the highest R^2^ scores (0.9984 and 0.9983), indicating superior prediction accuracy and model fit. In contrast, GLM showed the weakest performance, with the highest RMSE (0.3217 and 0.4212), MAE (0.265 and 0.347), and the lowest R^2^ values (0.812 and 0.822), suggesting it was less suited for capturing the complexity of the data. The performance of Gradient Boosting and KNN fell in the intermediate range, while SVM achieved relatively strong results but was still slightly outperformed by XGB in all metrics.

Based on the combination of evaluation results, XGB was selected as the optimal model with the highest R^2^ fitting capabilities with relatively low error metrics compared with the others, and therefore, we could leverage the XGB model for further model interpretation and predictive explanation. Slightly higher errors were also observed in the 400 bps data than 260 bps, which may reflect some increased noise from longer reads as well.

### Model evaluation on test set and cross-validation

To further evaluate model generalizability and stability, we assessed the XGB model on both a 30% Testing set and using 10-fold Cross-Validation. Table 1 summarizes the model performance metrics across two datasets (260 bps and 400 bps), reported using the evaluation metrics of MSE, MAE, and R^2^. The results demonstrated consistently high R^2^ values (more than 0.99%) and low error rates (less than 0.1 for both MSE and MAE) across both Testing and Cross-Validation strategies, suggesting robust predictive capability in both DNA 9-mer datasets of 260 and 400 bps. Notably, consistently high R^2^ values have indicated model robustness and stability, and the superior performance metrics for Cross-Validation also confirmed the model’s generalizability and minimized overfitting.

**Table 1.** Summary of test set and 10-fold cross-validation results for 260 bps and 400 bps datasets.

Dataset	Evaluation type	MSE	MAE	R^2^
260 bps mode, 9 mer, DNA, R10.4 flow cell	Test set (30%)	0.00050	0.01680	0.99910
	10-fold CV (average value)	0.00051	0.01702	0.99908
400 bps mode, 9 mer, DNA, R10.4 flow cell	Test set (30%)	0.00110	0.02548	0.99890
	10-fold CV (average value)	0.00109	0.02541	0.99891

Abbreviations: CV, cross-validation; MAE, mean absolute error; MSE, mean squared error; R^2^, coefficient of determination.

### Investigation of signal mechanisms according to nucleotides and positions

**Figure 2.**
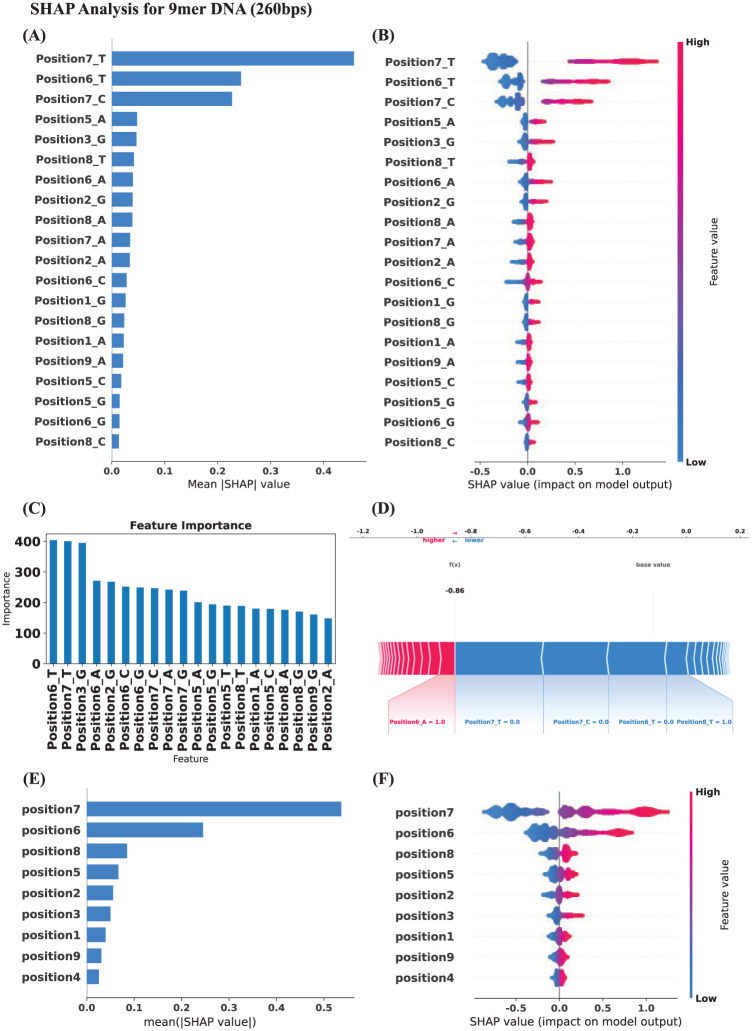
SHAP analysis plots and feature explanation results based on the XGB model for DNA 9mer ONT Database with 260 bp. (A) Absolute mean SHAP values showing contributing magnitude effects with descending order of bases and positions utilized. (B) SHAP values with magnitude and directions toward the base and positions utilized. Red colors represented larger feature values while the blue showing the minimal impact. (C) SHAP Feature Importance Analysis showing contributions of different variables in the construction of XGB model. (D) The Force Plot explaining the base values and detailed contribution processed of each base and position in the XGB model. (E) Absolute mean SHAP values showing contributing magnitude effects in descending order with only positional variables included. (F) SHAP values with magnitude and directions with only positional variables included.

To assess the positive or negative correlation between nucleotides, their positions, and electrical current levels, SHAP values were utilized to illustrate each feature’s impact on the model’s output. As shown in Figure 2A to C in the 260 bps dataset, the T base at the 7th position and the T base at the 6th position emerged as the most influential features, displaying the widest SHAP value distributions, indicating their significant impact on the model’s predictions. The SHAP values for the T base at the 7th position showed a consistent positive correlation with higher predictions, with higher values associated with higher electrical current levels. The T base at the 6th position exhibited a less pronounced effect than the 7th position, but still with a positive relationship toward ionic signals (Figure 2A to C).

Moreover, Figure 2E and F (260 bps) and Supplemental Figure 1E and F (400 bps) illustrated the impact of nucleotide positions—regardless of specific base identity—on the predicted electrical current. These plots have focused solely on how positional variation influences the model’s output. In both 260 bps and 400 bps, positions 7 and 6 showed the highest mean SHAP values with mainly positive directions, indicating strong positive contributions to current prediction. In addition, Figure 2D and Supplemental Figure 1D were the force plots to illustrate the changing effects and contributions from each base and position, and red and blue colors showed the higher or lower changes subsequently.

When considering the 400 bps dataset (Supplemental Figure 1A to C), similar trends were observed, with the T base at the 7th and the 6th position remaining the most influential features with positive correlations.

### Heatmap analysis for signal changes with different nucleotides

**Figure 3.**
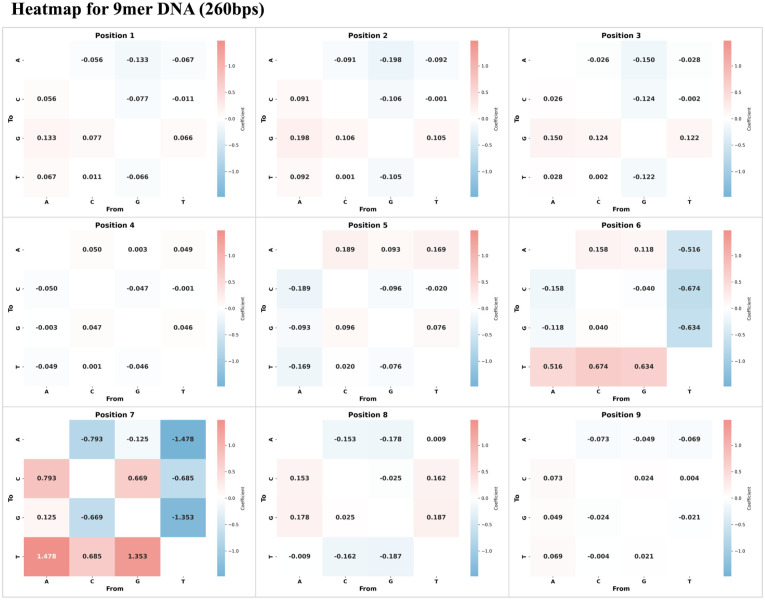
Heatmap showing the changes in signal coefficients with respect to transitions from original nucleotide (horizontal x-axis) to target nucleotide (vertical y-axis) in DNA 9mer ONT database with 260 bps.

The two heatmaps, for both datasets of the 260 bps (Figure 3) and 400 bps (Supplemental Figure 2), have revealed the effect of nanopore signals according to nucleotide transitions at each position of the 9-mer DNA sequence. Basic regression analyses with R^2^ = 0.812 and 0.822 for 260 bps and 400 bps were also computed to demonstrate the well-fitted performance for our transitional heatmap analysis.

Among all positions, positions 6 and 7 consistently exhibited the strongest transition effects, with clear symmetry between complementary transitions. For instance, at position 7, A→T and T→A transitions showed coefficients of +1.478 and −1.478 in the 260 bps dataset, increasing to +2.017 and −2.017 in the 400 bps dataset. Similarly, G→T and T→G at the same position 7 increased from ±1.353 to ±1.849, suggesting amplified signal sensitivity at higher sequencing speed. A similar trend was observed at position 6, where base changes A→T grew from +0.516 to +0.647, and G→T from +0.634 to +0.805, indicating that signal variation at this position was also enhanced under faster throughput. In addition, other positions, such as positions 5 and 9, which showed minimal impact in the 260-bps heatmap, displayed stronger effects in the 400-bps condition, with G→T at position 5 increasing from +0.076 to +0.145, with corresponding switches between the two datasets. These findings demonstrated that while positions 6 and 7 have consistently been the most impactful determinants, increased sequencing speed might amplify transitional effects at particular positions, highlighting the enhanced sensitivity of nanopore signal response and the importance of position-specific nucleotide transitions.

**Figure 4.**
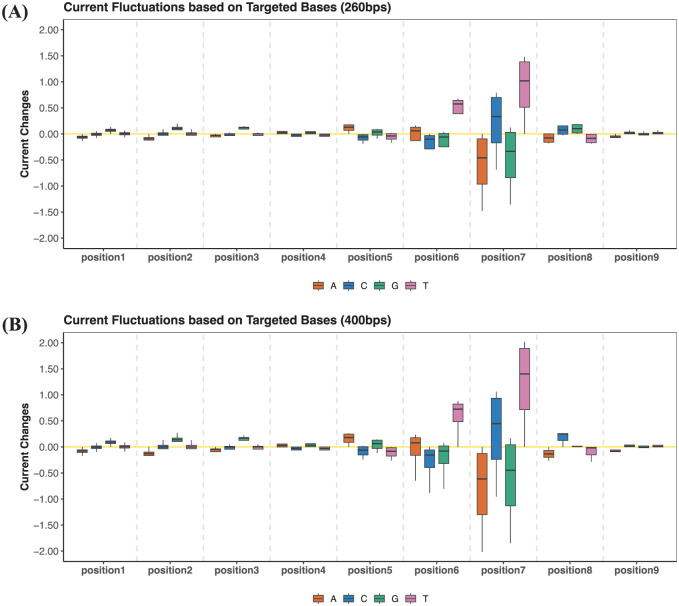
Box plots demonstrating nanopore current fluctuations due to transitions of bases, with one boxplot representing one targeted nucleotide, with analysis of DNA 9-mer ONT Dataset of 260 bps (A) and 400 bps (B). Positions 6 and 7 are the most important positions for determining the nanopore signals. The impact of nucleotides at positions 1, 2, 3, 4, and 9 is quite minimal.

Additional box plots were drawn to show the signal disturbance caused by the base changes from different positions, as vividly presented in Figure 4 (subplot A for 260 bps and subplot B for 400 bps). Four colors represented the targeted bases of A/C/G/T, and each box plot recorded all base changes with the same destination at a particular position. Specifically, positions 6 and 7 were found to have more apparent fluctuations than other positions, and the dataset of 400 bps seemed to have a larger range of transitional changes than the 260 bps, which were consistent with previous findings of the influential features. Moreover, the direction of such disturbance could also be clearly unveiled according to the signs of the changing statistics. Taking the most influential positions 6 and 7 as an example, base changes with targeted nucleotide of Thymine (T) would associate with positive increases in nanopore currents, while the Guanine (G) base labeled in green might correspond to the negative impact on signal changes.

## Discussion and Conclusion

This study has aimed to explore the relationship between nanopore ionic current signals and nucleotide types and positions using ONT 9-mer DNA sequences. Compared to prior commonly-applied 5-mer frameworks, our innovative investigation toward the 9-mer approach could offer richer sequence context and positional resolution with corresponding transitional studies.^7,28^

Based on our performance comparison and explainability enhancement using XGB and SHAP, we have identified positions 6 and 7, together with the nucleotide type of thymine (T), could mainly play the role of key contributors to nanopore signal variation. SHAP results also highlighted cytosine (C) nucleotide at positions 6–8, particularly the cytosine base at position 7, as an influential feature in both 260 bps and 400 bps datasets. Heatmap analysis also confirmed strong positional and base-specific effects, with positions of 6 and 7 being apparent in terms of transitions of bases and current fluctuations, along with the most pronounced impact of nucleotide T, especially at longer read lengths.

Our SHAP-based analysis revealed strong position-specific effects, with positions 6 and 7 of the 9-mer contributing most significantly to the predicted ionic current. This observation is consistent with prior biophysical studies indicating that nanopore signals are primarily influenced by a short window of nucleotides residing within the pore’s constriction zone.^2,9,29^ For example, Comer and Aksimentiev (2016) demonstrate that the ionic current is sensitive to the chemical identity and conformation of at least three continuous nucleotides,^30^ while Laszlo et al^9^ show that approximately four nucleotides within the MspA pore collectively modulate the current measurement.^31,32^ Notably, thymine (T) was identified as having a particularly strong impact on signal variation when located at these central positions in our analyses. This is also in line with prior computational findings that thymine-rich sequences tend to be associated with increased current, which might potentially be associated with thymine’s compact structure and lower polarizability to some extent.

While our study focuses on canonical DNA bases (A, T, C, G), the SHAP-based interpretability framework holds potential for extension to chemically modified bases. Previous studies have shown that modifications like 5-methylcytosine (5mC) can induce measurable changes in nanopore current signals,^33-35^ and applying our 9-mer contextual approach to such datasets could enable broader and more practical interpretation of epigenetic modifications.

With the purpose of constructing the preferred predictive models toward ionic current signals based on 9-mer DNA sequential context information, several models were designed on the same dataset with different fitting metrics evaluated. Different models have specific strengths with corresponding suitable situations to be applied. GLM extends traditional linear regression and serves as a baseline method, assuming a linear relationship between predictors and the response variable.^36^ DT models split the feature space into rule-based regions for interpretable predictions,^37^ while Gradient boosting iteratively combines weak learners to minimize prediction error.^38^ KNN is a nonparametric method that bases predictions on the most similar training samples,^39^ and SVM with a radial kernel could capture nonlinear relationships by projecting features into higher-dimensional space.^40^ XGB, an optimized ensemble method based on gradient boosting, is particularly efficient and offers feature importance scoring, which has been widely applied in bioinformatics due to its efficiency and accuracy.^41^ Its ability to handle structured input and provide interpretable feature importance makes it a promising tool for investigating nucleotide-level signal variation and detecting DNA modifications.

Our datasets have comprised mainly k-mer context, comprising both positional and base-specific information with one-hot coding. Such a tabular form of input and predictive regression purposes might possibly explain the excellent fitness of the XGB model, since such a tree ensemble model has demonstrated the outperformed fitting effects in terms of such tabular information.^42^ Although our model achieved near-perfect performance (~99%), we took several steps to ensure robustness by mitigating overfitting and evaluating generalizability. Specifically, we applied stratified data splitting to separate training and testing sets and validated performance across multiple cross-validation folds. In addition, the model was evaluated on two independent datasets generated at different sequencing speeds (260 bps and 400 bps), yielding consistently high accuracy and similar feature attribution patterns.

Despite the ideal results of high predictive accuracy in nanopore signal analysis and predictions, several challenges still remained due to the biological complexity of the sequencing process, and more future directions could be targeted.^2,14,43^ It should be admitted that factors such as DNA secondary structure, transient DNA-pore interactions, and experimental conditions could introduce subtle biases beyond the 9-mer sequence context. Future extensions incorporating more structural or contextual features may help address these limitations. Moreover, ionic current signals can be influenced by base modifications and RNA secondary structures, which are not yet fully incorporated into existing models.^43^ Future work may benefit from integrating biologically relevant features such as 8-oxoG and pseudouridine,^44,45^ as well as some common modifications of N^6^-methyladenine (m6A) and 5mC.^13^ Additional studies and structural analysis from RNA folding in replacement of the current DNA context might also be suggested.^46-48^ Furthermore, more adoption or addition of some DL architectures like CNNs^49^ or attention-based models,^50,51^ either with the entire framework or with a separate additive layer, might improve the model’s ability to detect more complicated sequence patterns. And even expanding the sequence window beyond 9-mers would also be a new trial, which might potentially enrich the contextual information and decode more interpretability toward longer sequences.

In conclusion, our studies have made innovative predictions from specific nucleotide bases and positions in determining the electrical current levels during the 9-mer DNA nanopore sequencing. The fitting results from XGB along with the SHAP tool, and heatmap figures, as well as transitional changes, have provided valuable insights into how nucleotide patterns affect the signals with detailed directions and magnitudes illustrated. These findings have targeted longer 9-mer sequences instead of commonly studied 5-mer sequences, showing important implications for decoding mechanisms behind nanopore signals with correlation detection of sequence context, which could be quite meaningful for analysis of the nanopore sequencing field.

## Supplemental Material

sj-docx-1-bbi-10.1177_11779322251378620 – Supplemental material for Understanding the Impact of Individual Nucleotide on Oxford Nanopore Current Signals With Interpretable Prediction Models Supplemental material, sj-docx-1-bbi-10.1177_11779322251378620 for Understanding the Impact of Individual Nucleotide on Oxford Nanopore Current Signals With Interpretable Prediction Models by Yenan Wang, Zhixing Wu and Jia Meng in Bioinformatics and Biology Insights
