# Supplementary material for: Understanding the Impact of Individual Nucleotide on Oxford Nanopore Current Signals With Interpretable Prediction Models
Source: Bioinform Biol Insights. 2025 Sep 22;19:11779322251378620. doi: 10.1177/11779322251378620 (PMC12457769; doi:10.1177/11779322251378620)
Supplement: sj-docx-1-bbi-10.1177_11779322251378620 – Supplemental material for Understanding the Impact of Individual Nucleotide on Oxford Nanopore Current Signals With Interpretable Prediction Models [file sj-docx-1-bbi-10.1177_11779322251378620.docx]

**Understanding the impact of individual nucleotide on Oxford nanopore current signals with interpretable prediction models**

**Supplementary Materials**

**Supplementary Figure 1** SHAP analysis plots and feature explanation results based on XGB model for DNA 9mer ONT Database with 400bp. **(A)** Absolute mean SHAP values showing contributing magnitude effects with descending order of bases and positions utilized. **(B)** SHAP values with magnitude and directions towards the base and positions utilized. Red colors represented larger feature values while the blue showing the minimal impact. **(C)** SHAP Feature Importance Analysis showing contributions of different variables in the construction of XGB model. **(D)** The Force Plot explaining the base values and detailed contribution processed of each base and position in the XGB model. **(E)** Absolute mean SHAP values showing contributing magnitude effects in descending order with only positional variables included. **(F)** SHAP values with magnitude and directions with only positional variables included.

**Supplementary Figure 2** Heatmap showing the changes in signal coefficients with respect to transitions from original nucleotide (Horizontal x-axis) to Target nucleotide (Vertical y-axis) in DNA 9mer ONT Database with 400bps.
